# Mass Spectrometry-Based Bacterial Proteomics: Focus on Dermatologic Microbial Pathogens

**DOI:** 10.3389/fmicb.2016.00181

**Published:** 2016-02-19

**Authors:** Youcef Soufi, Boumediene Soufi

**Affiliations:** ^1^College of Medicine, University of Manitoba, WinnipegMB, Canada; ^2^Independent Academic ScholarMagdeburg, Germany

**Keywords:** mass spectrometry, proteomics, pathogenic bacteria, skin disease, dermatology, bacterial resistance

## Abstract

The composition of human skin acts as a natural habitat for various bacterial species that function in a commensal and symbiotic fashion. In a healthy individual, bacterial flora serves to protect the host. Under certain conditions such as minor trauma, impaired host immunity, or environmental factors, the risk of developing skin infections is increased. Although a large majority of bacterial associated skin infections are common, a portion can potentially manifest into clinically significant morbidity. For example, Gram-positive species that typically reside on the skin such as *Staphylococcus* and *Streptococcus* can cause numerous epidermal (impetigo, ecthyma) and dermal (cellulitis, necrotizing fasciitis, erysipelas) skin infections. Moreover, the increasing incidence of bacterial antibiotic resistance represents a serious challenge to modern medicine and threatens the health care system. Therefore, it is critical to develop tools and strategies that can allow us to better elucidate the nature and mechanism of bacterial virulence. To this end, mass spectrometry (MS)-based proteomics has been revolutionizing biomedical research, and has positively impacted the microbiology field. Advances in MS technologies have paved the way for numerous bacterial proteomes and their respective post translational modifications (PTMs) to be accurately identified and quantified in a high throughput and robust fashion. This technological platform offers critical information with regards to signal transduction, adherence, and microbial–host interactions associated with bacterial pathogenesis. This mini-review serves to highlight the current progress proteomics has contributed toward the understanding of bacteria that are associated with skin related diseases, infections, and antibiotic resistance.

## Introduction

Skin has a primary role to act as a physical barrier in order to protect the body from temperature variations, microorganisms, or toxic substrates. While *in utero* skin is sterile, after birth it becomes rapidly colonized with numerous microorganisms which aids toward protecting the body (Capone et al., 2011). Bacterially derived dermatologic conditions can range from relatively benign conditions (assuming non-immunocompromised patients) such as cellulitis, erysipelas, folliculitis, or impetigo to serious clinical morbidity, such as necrotizing fasciitis (Stulberg et al., 2002). Underlying medical conditions such as diabetes mellitus or AIDS are examples where commensal bacteria can invade the skin resulting in infections that can manifest from mild to the life threatening (Swartz, 2004).

In recent years there have been growing concerns with respect to the challenges faced in the emergent inability to provide effective therapeutic interventions for patients with pathologies of bacterial etiology, due to increasing rates of antibiotic resistance. Considering the ease of transmission with respect to resistance genes amongst mucosal and epidermal flora, special consideration should be given to dermatologic related bacterial conditions (Espersen, 1998).

Mass spectrometry (MS)-based proteomics has the capability to study proteins and their interactions in order to better understand dysregulations in infection disorders (List et al., 2008), reveal antibiotic resistance mechanisms (Lee et al., 2015) and significant new targets for future drug discovery. Current MS-based proteomics technologies have advanced to the point where they are amenable to any biological system. With regards to bacterial organisms, they are particularly attractive models to apply proteomics based approaches due to their smaller proteomes and modifications compared to eukaryotes allowing for comprehensive proteome coverage (Stekhoven et al., 2014).

## Skin Microbiome

The skin is host to many different bacterial species, fungi, viruses and mites. Most of the bacteria fall into four phyla: Actinobacteria, Firmicutes, Bacteroidetes, and Proteobacteria from which *Propionibacterium, Corynebacterium, Staphylococcus, Micrococcus, Streptococcus*, and *Brevibacterium* are highly abundant (van Rensburg et al., 2015) Gram-positive bacteria, since Gram-negative members typically cannot cope with the relatively dry environment of healthy human skin (Del Rosso and Leyden, 2007). In certain physiological conditions where the skin becomes moist, some Gram-negative bacteria can colonize such as *Acinetobacter* sp. (Del Rosso and Leyden, 2007). The diverse community of the skin, known as the skin microbiome, is defined by host physiology, host genotype, immune system, environment, and lifestyle (Grice and Segre, 2011). However, most of these microorganisms are harmless and many bacterial species act in a commensal fashion by which each species benefits through the exchange of nutrients as well as protect the host from pathogens without negatively affecting each other (Mankowska-Wierzbicka et al., 2015). Knowledge toward the bacterial microbiome is mainly derived from the conventional culture-based approaches, although in recent years DNA sequencing technologies combined with bioinformatic analysis and metagenomics approaches have allowed comprehensive examination of microbial communities (Kuczynski et al., 2012), yet uncertainties remain as to what defines a “normal” microbiome (Backhed et al., 2012).

The underlying contribution of the microbiome in the clinical picture of skin disorders is clear from the perspective of antibacterial treatment, however, the molecular dynamics between the microbiome and host remains largely unknown. Disturbances in homeostasis between microbiome and host can manifest into skin disorders such as atopic dermatitis (AD) or psoriasis (Kuo et al., 2013). Both disorders are connected with dysregulation of the skin immune response. However, while AD lesions are characterized by low level, psoriatic lesions are characterized by high level of antimicrobial peptide production (Nomura et al., 2003). Additionally, AD lesions are regularly infected with microbial pathogens.

The bacterial genus of *Staphylococcus* sp. is composed of approximately 40 different species both commensal (unable to produce virulence factor coagulase) and pathogenic, and play an important role in skin health and pathology (Del Rosso and Leyden, 2007). For example, *Staphylococcus aureus* is a medically relevant bacterial pathogen that is associated with the increasing rate of antibiotic resistance mechanisms as well as systemic and cutaneous infections (Becker et al., 2007), including AD (Hanifin and Rogge, 1977). However, it was previously thought that *S. aureus* causes AD alone, a recent study showed that the skin microbiome composition has a temporal change and it depends on disease flares and treatment. In the active state of the disease, the abundance of *S. aureus* and skin commensal *S. epidermidis* was increased, while *Streptococcus, Propionibacterium*, and *Corynebacterium* were increased following therapy (Kong et al., 2012). Increased abundance of *S. epidermidis* could reflect a microbial response to overgrowth of *S. aureus*, since it can selectively inhibit *S. aureus* growth (Iwase et al., 2010; Zeeuwen et al., 2013). Moreover, a recent study identified the presence of a distinct microbiome capable of direct communication with the host at the sub-epidermal compartments of the skin, an area previously thought to be sterile (Nakatsuji et al., 2013). While these types of studies employing different techniques have characterized the bacterial species that make up the skin microbiome, proteomics is required in order to identify proteins and their respective pathways involved during these interactions, which could directly contribute to the better understanding of the complex interplay between microbiome and host.

## Shotgun MS-Based Proteomics

Due to the progress made toward state of the art next generation sequencing methodologies applied to generate fully annotated genomes, the number of fully sequenced microbial genomes has increased dramatically (Ribeiro et al., 2012). This has enabled state of the art MS-based proteomics technologies to rapidly advance in order to study microbial models and their communities in a robust and systematic fashion.

Two-dimensional polyacrylamide gel electrophoresis (2D-PAGE), followed by MS analysis has served as the main proteomics method of choice in the past and has been utilized in many skin associated bacterial pathogen studies (Kohler et al., 2005; Becher et al., 2009; Francois et al., 2014). In this technique, proteins are separated according to their molecular weight and isoelectric point migrating and accumulating as protein spots on the gel matrix. Although this approach can resolve multiples of thousands of proteins, it does carry some disadvantages especially with respect toward the identification of low abundant proteins, proteins with extremely high and low molecular weights, and is rather impractical with respect to large scale site specific analysis of post translational modifications (PTMs) such as phosphorylation.

**FIGURE 1:**
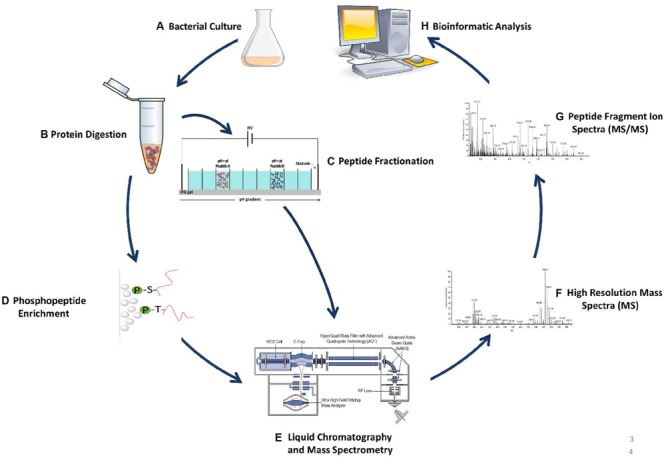
**Example of shotgun proteomics workflow.** This workflow provides a blueprint that can decode protein dynamics from complex crude protein extracts toward elucidation of numerous novel biological functions. **(A)** Cells are grown in culture or other growth models, and lysed using various procedures. **(B)** Protein extracts are digested for example by trypsin to generate peptides. **(C)** Peptides are fractionated in order to decrease peptide complexity which increase both identification and quantification rates upon MS analysis. **(D)** If PTM analysis is required, various enrichment strategies can be performed such as titanium chromatography in the case of phosphopeptide enrichment. **(E)** Resulting peptide extracts are separated on nanoflow HPLC and measured on a high resolution mass spectrometer (e.g., Thermo Scientific Q Exactive HF). **(F)** Relative peptide quantitation (full-scan MS). **(G)** Peptide identification through sequencing and detection to acquire MS/MS spectra. **(H)** Data processing utilizing numerous bioinformatic approaches.

The inherent limitations associated with 2D-PAGE MS lead to the development of gel-free MS-based approaches better known as “shotgun” proteomics. Advances made in shotgun based proteomics approaches is largely due to technological improvements made in high performance mass spectrometers. Since most biological samples consist of very complex peptide mixtures, mass spectrometers must be capable of ensuring a deep analytical coverage while maintaining a high level of robustness, sensitivity, and measurement accuracy (Mann and Kelleher, 2008). Newer generations of hybrid mass spectrometers such as but not limited to the LTQ-Orbitrap (Hu et al., 2005), LTQ Orbitrap Velos (Olsen et al., 2009), Q-Exactive HF (Scheltema et al., 2014), and Orbitrap Fusion (Erickson et al., 2015) can achieve precise mass accuracy at very high acquisition speeds, and resolution which allows for a complete sampling of complex peptide extracts providing comprehensive proteome coverage. Combined with high performance liquid chromatography (HPLC) technologies, these LC-MS workflows also referred to as gel-free shotgun proteomics allow for the quantification and identification of entire proteomes as well as their protein modifications across different biological samples. An overview of a typical shotgun MS-based experimental workflow is illustrated in **Figure 1**.

For example, a similar approach as described above was applied to the bacterium *Propionibacterium acnes* (Bek-Thomsen et al., 2014). *P. acnes* is known to be associated with the inflammatory condition of the sebaceous human follicles known as acne vulgaris (Williams et al., 2012). The authors utilized proteomics in order to identify both human and *P. acnes* proteins using sebaceous follicular casts. To this end, many proteins involved in wound healing, inflammation, and tissue formation were identified. The most abundant *P. acnes* proteins were CAMP factors, and surface exposed dermatan sulfate adhesins. This study is the first of its kind in terms of analyzing the proteomes of both humans and bacteria on sebaceous follicular casts and demonstrates the importance of employing shotgun based proteomics workflows (Bek-Thomsen et al., 2014).

A recent study employed a combinatorial approach utilizing 16SrRNA sequencing with ultra-performance liquid chromatography/quadrupole time of flight (UPLC-TOF) MS toward the generation of a 3-dimensional topographical map of microbes and their molecules of origin (peptides, metabolites) distributed on the surface of the human skin (Bouslimani et al., 2015). This information was utilized to identify microbial species present and correlated with the chemical environment of the skin and serves as a powerful approach toward elucidating how the microbiome interacts and subsequently modifies different areas of the human skin in a species specific fashion.

## Shotgun MS-Based Quantitative Proteomics

The complete identification of a proteome and its respective PTMs provides a better understanding toward biological function and regulation. However, it is equally important to quantify dynamics of proteins relative to each other under different biological conditions, disease states, and perturbations. This information can be obtained through the use of quantitative proteomics of which numerous approaches have been developed that are compatible with shotgun MS-based proteomics strategies.

One common approach is to introduce a stable non-radioactive isotope label either chemically or metabolically on the peptide or protein level. Through this technique, the relative intensities of peptides are measured, via MS thus identifying and quantifying regulated proteins from different samples through the obtained mass spectra. Examples of such techniques include but they are not limited to Stable Isotope Labeling by Amino acids in Cell culture (SILAC; Ong et al., 2002) or ^15^N labeling (Hempel et al., 2010; Soufi et al., 2015), both of which have been successfully implemented in a variety of bacteria (Soufi et al., 2010, 2015; Hempel et al., 2011; Soares et al., 2013; Misra et al., 2014; Soufi and Macek, 2014; Boysen et al., 2015). Furthermore, an innovative MS-based technique known as cell type-specific labeling using amino acid precursors (CTAP) has the ability to continuously label the proteome of individual cell types actively growing in a co-culture environment, allowing for the elucidation of the “cell-of-origin” of proteins in multicellular environments (Gauthier et al., 2013). Although this method was demonstrated in eukaryotic models, the application toward bacteria especially those in multispecies environments, such as human microbiomes, biofilms, etc. could significantly contribute toward the better understanding of the dynamics between different bacterial species inhabiting the same environment.

Although metabolic labeling approaches such as SILAC are considered by many to be the most accurate method toward global protein quantitation (Ong et al., 2002), they may be difficult to implement due to technical challenges with incorporation of the chemical or metabolic labeling approach, or challenges posed in complex biological models such as those involving the skin. Therefore, due to rapid advances made in LC-MS, label free quantitation (LFQ) approaches are now possible and routinely performed (Neilson et al., 2011). In this approach, different peptide samples are measured via MS and compared either by the total number of sequenced (MS/MS) spectra or the total extracted ion currents under controlled conditions.

The LFQ methodology was successfully applied to study the role of *S. aureus* in patients with ectodermal dysplasia and AD (Burian et al., 2015). Patients suffering from AD are highly correlated with the presence of opportunistic *S. aureus* infections due to having a reduced immune response, and have been shown to contain a lower amount of peptides originating from the natural antimicrobial dermcidin found in sweat leading to decreased levels of antimicrobial activity (Schittek, 2011). The authors proved through proteomics analysis of the secretome of diseased vs. healthy patients that a similar mechanism of reduced sweat derived dermcidin also exists in patients with ectodermal dysplasia, making these individuals highly prone to acquiring *S. aureus* infections (Burian et al., 2015).

Quantitative proteomics techniques can be utilized to estimate the absolute molar amount or concentration of a particular protein per cell. This information is highly relevant especially within the clinical context (Rodriguez-Suarez and Whetton, 2013). Many variants of this technique exist and all involve spiking in a known concentration of an internal protein standard into the sample, followed by MS analysis, and comparing resulting sample peptide measurements to the internal standard. Techniques include FLEXIQuant (Singh et al., 2009), absolute quantification using protein epitope signature tags (PrEST; Zeiler et al., 2012), intensity based absolute quantification (iBAQ; Schwanhausser et al., 2011), and absolute quantification (AQUA; Gerber et al., 2003). These methods can be applied to bacteria and serve as an important tool toward understanding the underlying mechanisms during bacterial virulence and antibiotic resistance.

## Post Translational Modifications (PTMS)

Various PTMs on bacterial proteins such as phosphorylation, acetylation, methylation, and deamidation serve as an efficient means of controlling signal transduction, virulence and regulatory processes. PTMs represent a significant process in the life cycle of bacteria and can modulate key virulence factors and are attractive targets for novel therapies.

Detecting these PTMs in bacteria poses a technical challenge due to the fact that they are difficult to discover as these modifications typically exist at low levels of abundance. To circumvent this issue, specific enrichment strategies targeting certain PTMs can be utilized in order to decrease peptide complexity thereby increasing the likeliness of detection and subsequent characterization. For example, immunoaffinity enrichment is typically employed to select for lysine acetylated peptides (Rardin et al., 2013). Moreover, similar enrichment strategies are employed to capture phosphorylation events on serine, threonine, and tyrosine (S/T/Y) amino acid residues. These PTMs were once thought to exist solely in eukaryotes, however, MS-based proteomics combined with phosphopeptide enrichment strategies such as titanium dioxide chromatography (TiO_2_; Pinkse et al., 2004), immunoprecipitation (Rush et al., 2005) or immobilized metal ion affinity chromatography (IMAC; Villen and Gygi, 2008) have now established S/T/Y phosphorylation as a frequent and important PTM among different bacterial species (Macek et al., 2007, 2008; Soufi et al., 2008; Lin et al., 2009; Sun et al., 2010; Manteca et al., 2011; Misra et al., 2011; Cousin et al., 2013).

A recent shotgun based high resolution LC-MS/MS approach involving the enrichment of surface proteins using “trypsin shaving” was applied to the opportunistic pathogen *S. aureus* toward the identification of hydroxymethylation on aspargine and glutamine amino acid residues in an attempt to identify the presence and potential regulatory importance of this PTM on surface proteins which are known to assist in the colonization and invasion of the host cell (Waridel et al., 2012). The authors reported a total of 15 proteins (mostly surface proteins) that contained hydroxymethylation modifications and could play a role in virulence factor modulation.

*Pseudomonas aeruginosa*, an opportunistic bacterial pathogen, is associated with immunocompromised patients and nosocomial infections. *P. aeruginosa* infections can enter the body through the skin, and can be life threatening due to their ability to develop antibiotic resistance (Ouidir et al., 2015). A study involving *P. aeruginosa*, employed an effective combinatorial method of immunoaffinity assays, complex peptide fractionation, and shotgun proteomics toward the identification and characterization of lysine acetylation, a reversible PTM that has recently been implicated as an important regulatory mechanism in many bacteria (Yu et al., 2008; Kim et al., 2013; Zhang et al., 2013; Liao et al., 2014). This approach lead to the identification of 320 acetylated proteins in a wide variety of functional classes. The study also identified novel lysine acetylation events in virulence factors known to assist in host immune response evasion such as chitin binding protein, serine protease, exotoxin A, and hemolysin which potentially implies that lysine acetylation events in *P. aeruginosa* plays a role in mechanisms involving virulence (Ouidir et al., 2015).

Cysteine phosphorylation in *S. aureus* was shown to assist in the regulation of bacterial virulence and vancomycin resistance (Sun et al., 2012). Utilizing high resolution MS, the authors elucidated in a site specific fashion, that cysteine phosphorylation events occurred in various proteins many of which are global regulators that control important biological processes (Sun et al., 2012). Moreover, the eukaryotic-like Ser/Thr kinase and phosphatase pair Stk1/Stp1 was found to regulate cysteine phosphorylation in many Gram-positive bacteria providing an important piece of information toward the underlying regulatory mechanism of these events (Sun et al., 2012).

## Conclusion and Future Outlook

A wide range of dermatological microbial associated diseases presents current and future challenges to health care providers. While in the last decade metagenomics data provided a higher level of understanding of the microbial skin environment, understanding signal transduction, virulence, regulatory processes, and dynamics between different bacterial species is essential in order to improve the overall standards and quality of patient care and treatment. MS-based proteomics has the capability to provide this knowledge. To this end, a ground-breaking endeavor with proteomics at the forefront is required in order to elucidate the specific mechanisms involved in skin infections, bacterial resistance as well as the complex microbiome and host relationship.

## Author Contributions

YS wrote the manuscript. BS assisted with writing of the manuscript and generated the figure.

## Conflict of Interest Statement

The authors declare that the research was conducted in the absence of any commercial or financial relationships that could be construed as a potential conflict of interest.
